# Identification of Granite Varieties from Colour Spectrum Data

**DOI:** 10.3390/s100908572

**Published:** 2010-09-14

**Authors:** María Araújo, Javier Martínez, Celestino Ordóñez, José Antonio Vilán

**Affiliations:** 1 Department of Environmental Engineering, University of Vigo, Vigo 36310, Spain; E-Mail: maraujo@uvigo.es (M.A.); 2 Academia General Militar, Centro Universitario de la Defensa, Zaragoza 50090, Spain; E-Mail: javier.martinez@uvigo.es (J.M.); 3 Department of Mechanical Engineering, University of Vigo, Vigo 36310, Spain; E-Mail: jvilan@uvigo.es (J.A.V.)

**Keywords:** spectrophotometer, functional data, classification, SVM, PUK kernel

## Abstract

The granite processing sector of the northwest of Spain handles many varieties of granite with specific technical and aesthetic properties that command different prices in the natural stone market. Hence, correct granite identification and classification from the outset of processing to the end-product stage optimizes the management and control of stocks of granite slabs and tiles and facilitates the operation of traceability systems. We describe a methodology for automatically identifying granite varieties by processing spectral information captured by a spectrophotometer at various stages of processing using functional machine learning techniques.

## 1. Introduction

A current trend in granite processing plants located in northwest Spain is the implementation of traceability systems to better control and manage stocks of slabs and end products resulting from the processing of a wide range of lithological materials, all considered as granite varieties from a commercial perspective. Cut and processed blocks have different mineralogical characteristics and origins, and this enormously complicates control over end products. Blocks are initially identified in the quarry by marks indicating type and origin and the edges of slabs, once sawn, are colour coded for identification purposes.

The market, however, demands end products of specific sizes and shapes (square and rectangular) and finishes with arrises and perpendicular edges. Consequently, the marks made on the edges of slabs are inevitable lost when these are cut using a diamond-disk saw.

We describe an expert system for identifying different types of granite by spectrophotometer-based colour characterization applied in all the processing phases until perfectly shaped and squared slabs are obtained, with the ultimate aim of improving the current discontinuous control and management system used in plants. The effectiveness of this approach in terms of analysing and characterizing stone types on the basis of colour has already been reported by other authors [1–4].

The classical methodology for classifying and identifying different varieties of granite is to analyse textural aspects in direct petrography studies of thin laminates. Other approaches are to study photomicrographs of rock in thin sections using digital image processing and texture analysis [5], to analyse images for colour and texture attributes [6,7] and to make quantitative colour measurements from scanner-captured digital images [8].

Our identification methodology is based on: (1) objectively characterizing stone colour using a spectrophotometer; (2) discretely transforming reflectance data (collected by the spectrophotometer sensors in various sections of the visible-light region) into spectral curves in a smoothing process; and (3) resolving the classification problem using machine learning techniques for functional data. Our approach ensures objectivity and minimizes possible human error in the identification process associated with different perceptions of colour, observation times and object sizes.

The functional spectral information was processed using a functional linear regression model and a functional support vector machine (SVM) with a PUK kernel (see [9,10] for classification problems successfully solved using SVMs with a PUK kernel and [4,11–13] for functional problems resolved by SVMs and functional linear regression).

The article is laid out as follows: Section 2 describes the theory underlying functional classification techniques used to handle spectral information collected by a spectrophotometer, Section 3 details both the methodology for automated granite rock identification and the stone processing phases to be integrated in the system. Section 4 describes the data processing results obtained for each implemented algorithm. Finally, Section 5 describes our main conclusions.

## 2. Mathematical Models

### 2.1. Functional Data Analysis

The resolution of classification, regression and principal component problems using statistical techniques is typically scalar or vectorial. The analysis of functions assumes a finite set of values [14], that is, the problem is vectorial. By making the problem a functional one, the entire set of data can be evaluated and analysed and this allows variations in the function to be analysed (for example, in a temporal process) by studying the different functional derivatives. Functional data analysis (FDA) was a technique first developed by Deville [15] and subsequently further refined by Ramsay and Silverman [14] for the purpose of resolving problems whose data was possibly functional in nature.

In FDA, the first step is to perform smoothing to fit curves to a set of functional data. This process is described immediately below and the rest of the section describes the two FDA techniques used in our research to identify granite varieties from surface colour.

### 2.2. Smoothing

Given a set of observations *x*(*t_j_*) in a set of *n_p_* points, where *t_j_* ∈ *R* represents each instant of time, let *x*(*t*) ∈ *χ* ⊂ *F* be a set of discrete observations of the function, where *F* is a functional space. To estimate the function *x*(*t*), let F = *span* {*φ*_1,...,_*φ_n_b__*}, where {*φ_k_*}, with *k* = 1,...., *n*, is a set of basis functions. In view of this expansion:

(1) $$x(t)\;=\;\sum_{k=1}^{n_{b}}c_{k}\phi_{k}(t)$$

where *c_k_*, *k* = 1,....*n_b_* represent the coefficients of the function *x*(*t*) with respect to the basis functions.

The smoothing problem now consists of determining the solution to the following regularization problem:

(2) $$\underset{x\in F}{\text{min}}\;\sum_{j=1}^{n_{p}}{\left\{z_{j}-x\left(t_{j}\right)\right\}}^{2}\;+\;\lambda \Gamma (x)$$

where *z_j_* = *x*(*t_j_*) + *ε_j_* is the result of observing *x* at point *t_j_*, Γ is an operator that penalizes the complexity of the solution and *λ* is the regularization parameter. Bearing in mind this expansion, the regularization problem can be written as:

(3) $$\underset{c}{\text{min}}\;\left\{{\left(z\;-\;\Phi c\right)}^{T}\;(z\;-\;\Phi c)\;+\;\lambda c^{T}\;Rc\right\}$$

where **z** = (*z*_1_,...,*z_n_p__*)*^T^* is the vector of observations subject to noise, **c** = (*c*_1_,..., *c_n_b__*)*^T^* is the vector of coefficients for the functional expansion, *λ* is the regularization parameter, **Φ** is the *n_p_* × *n_b_* matrix with elements **Φ***_jk_* = *φ_k_* (*t_j_*), and **R** is the *n_b_* × *n_b_* matrix with elements as follows:

(4) $$R_{\mathrm{kl}}\;=\;{\langle D^{2}\varphi_{k},D^{2}\varphi_{l}\rangle }_{L_{2}(T)}\;=\;\int_{T}D^{2}\varphi_{k}(t)D^{2}\varphi_{l}(t)\mathrm{dt}$$

where *D*^2^ is the second-order differential operator.

Of possible families of basis functions, we can mention the polynomials, the splines and, in the specific case of the Fourier family of functions, orthonormal basis functions, where the matrix **R** is an identity matrix.

### 2.3. Functional Linear Regression

The classic formulation of a linear regression model is given as:

(5) y = Xb + δ

where **y** is typically a vector of observations, **X** a matrix that defines a linear transformation from parameter space to observation space, **b** is the vector of regression model coefficients and *δ* is a Gaussian-type error with zero mean. Making the matrix **X** and the vector **b** functional, this model can be extended to functional linear regression for scalar responses [14] as follows:

(6) $$y\;=\;\int_{0}^{T}x(s)\beta (s)\mathrm{ds}\;+\;\delta$$

where the function *β*(*s*) is determined using the minimum squared error criterion [14].

To estimate the function *β*(*s*), we used functional decomposition for finite-dimension spaces [14], which, to reduce the degrees of freedom of the regression, performs regularization using basis functions as denoted in (1). Hence, with **φ** as a vector of basis functions for length *n_β_*, we have:

(7) $$\beta (s)\;=\;\sum_{k=1}^{n_{\beta }}d_{k}\varphi_{k}(s)\;=\;\varphi^{T}\;(s)d$$

Each observed function can be expressed as a function of other basis functions **ψ**:

(8) $$x_{i}(s)\;=\;\sum_{k=1}^{n_{\beta }}c_{\mathrm{ik}}\psi_{k}(s)\;=\;c_{i}^{T}\psi (s)\;\Rightarrow \;x(s)\;=\;C\psi (s)$$

where **C** is the matrix of coefficients of the functional input variables **x**(*s*) with regard to the chosen basis functions **ψ**

Therefore, the prediction **ŷ** can be expressed as follows:

(9) $$\hat{y}\;=\;CJ_{\varphi \psi }d$$

where **J***_φψ_* is a matrix expressed as follows:

(10) $$J_{\phi \psi }\;=\;\int \psi (s)\phi^{T}(s)\mathrm{ds}$$

In a classification problem, the response variable takes values in a finite set of values, *y* ∈ {1,2,..., *c*} where *c* is the number of classes presented by the problem. The above regression problem, in which the response variable can take values in an infinite set of values, has been adapted to the classification problem by approximating the numeric value provided by the model to the nearest category or class.

### 2.4. Support Vector Machines for Functional Data

SVMs for classification [16] are essentially the result of implementing a linear classification rule that maximizes the distance between classes (maximum margin) in a larger dimension space that is the result of suitably transforming the input space. This linear classifier in the new space gives rise to a non-linear classifier with an arbitrary degree of complexity in the original input space.

Given a typical classification problem of two classes and a sample of data ${\left\{\left(x_{i},y_{i}\right)\right\}}_{i=1}^{n_{b}}$ with *y_i_* ∈ {−1,1} and *x_i_* ∈ *X*, with *X* an arbitrary Hilbert space, the SVM is the solution to the following problem:

(11) $$\begin{array}{c} \underset{w,b,\xi }{\text{min}}\frac{1}{2}\left\{\left\|w^{2}\right\|\;+\;C\sum_{i=1}^{n_{b}}\xi_{i}\right\}\\ \left(\begin{array}{c} y_{i}\left(\langle w,\psi \left(x_{i}\right)\rangle \;+\;b\right)\;\geq \;1\;-\;\xi_{i}\\ \xi_{i}\;\geq \;0 \end{array}\right)i\;=\;1,...,n \end{array}$$

where *ξ_i_* are slack variables which serve to admit a series of poorly classified observations (that is, a soft margin) and the parameter *C* expresses the importance assigned to these poorly classified cases. *ψ : X* → *Z* is a transformation of the input space into a new space *Z* usually of larger dimension, where an inner product is defined by means of a positive definite function *k* (kernel):

(12) $$\langle \psi \left(x\right),\psi \left(x^{\prime }\right)\rangle \;=\;\sum_{i}\psi_{i}\left(x\right)\psi_{i}\left(x^{\prime }\right)\;=\;k\left(x,x^{\prime }\right)$$

The above problem is quadratic with linear constraints, and so the Kuhn-Tucker optimality conditions are necessary and sufficient. The solution, which can be obtained from the dual problem, is a linear combination of a subset of sample points denominated support vectors (s.v.) as follows:

(13) $$\begin{array}{lll} w & = & \sum_{\text{s.v.}}\beta_{i}\psi (x_{i})\;\Rightarrow \\ f_{w,b}(x) & = & \sum_{\text{s.v.}}\beta_{i}\langle \psi (x_{i}),\psi (x)\rangle \;+\;b\;=\;\sum_{\text{s.v.}}\beta_{i}k\left(x_{i},x\right)+b \end{array}$$

The classification rule is ŷ(*x*) = *h*(*x*) = *sign*(*f_w,b_*(*x*)). Consequently, specific knowledge is not required regarding how the non-linearity of the solution was obtained, nor is it necessary to calculate the inner product; the kernel *k* itself is sufficient to determine the solution.

If the input space is included in a functional Hilbert space spanned by a set of basis functions, *X* ⊂ *F* = *span*{*φ*_1_,...,*φ_n_b__*}, the functional version of the SVM for classification is obtained [17]. In this case:

(14) $$w\;=\;\sum_{\text{s.v.}}\beta_{i}x_{i}\;=\;\sum_{\text{s.v.}}\beta_{i}\sum_{k}x_{i}^{k}\varphi_{k}\;=\;\sum_{k}(\sum_{\text{s.v.}}\beta_{i}x_{i}^{k})\varphi_{k}\;\in \;F$$

is also a function in *F*with coefficients $w_{k}=\Sigma_{\text{s.v.}}\beta_{i}x_{i}^{k}$, where $x_{i}=\sum_{k}x_{i}^{k}\varphi_{k}$ is the expression of each element of the sample in terms of the basis functions.

If the kernel has the general form *k*(*x*, *x*′) = *κ*(〈*x*, *x*′〉), then:

(15) $$\begin{array}{ll} f_{w,b}(x) & =\sum_{\text{s.v.}}\beta_{i}k\left(x_{i},x\right)+b=\sum_{\text{s.v.}}\beta_{i}\kappa \left(\langle x_{i},x\rangle \right)+b\\ & =\sum_{\text{s.v.}}\beta_{i}\kappa \left(\sum_{k}\sum_{l}x_{i}^{k}x^{l}\langle \varphi_{k},\varphi_{l}\rangle \right)+b=\sum_{\text{s.v.}}\beta_{i}\kappa \left(x_{i}^{T}\Phi x\right)+b \end{array}$$

where *x* = ∑*_k_x^k^φ_k_*, **x** and **x***_i_* are the vectors of coefficients for the functions *x* and *x_i_*, respectively, and **Φ** is the matrix with elements Φ*_kl_* = 〈*φ_k_*, *φ_l_*〉.

Of the many possibilities for selecting the kernel function [18], for this study we used the universal Pearson VII function [19] as the kernel (PUK), as it is more flexible for linear, polynomial and Gaussian functions [20]. This kernel function, which has been recently used by authors in different fields [9,10], is formulated as follows:

(16) $$g(x)=\frac{H}{{\left[1+{\left(\frac{2(x-x_{0})\sqrt{2^{\left(1/\omega \right)}-1}}{\sigma }\right)}^{2}\right]}^{\omega }}$$

where *H* is the height at the centre *x*_0_ of the peak, and *x* represents the independent variable.

The parameters *σ* and *ω* control the half-width (also called the Pearson width) and the tailing factor of the peak. The main reason for using the Pearson VII function is its flexibility: varying the parameter *ω* changes a Gaussian shape (*ω* approximates infinity) to a Lorentzian shape (*ω* equals 1) [20].

## 3. Identification Methodology for Granite Varieties

### 3.1. Data Pre-processing

The colour of granite varieties was characterized using a colour reflectance measurement instrument. As well as showing numeric information on colour in standard colour spaces (CIE L*a*b* [21,22]), it provided information on spectral reflectance by capturing the light reflected by each sample in each of the wavelength bands of the visible spectrum considered.

The spectral information of the stone was reflected as a set of discrete points by a spectrophotometer. Parameters were specifically configured to enable optimal capture of the colour peculiarities of each sample of granite analysed.

**Figure 1.**
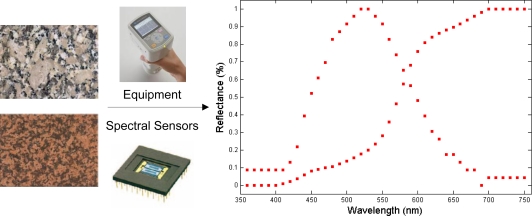
Vectorial spectral information captured for two granite specimens for 40 points corresponding to the visible area of the spectrum.

A Konica-Minolta CM-700d/600d spectrophotometer was used, equipped with CM-S100w SpectraMagic NX software, D65 illuminant, 10° observer and target diameter 8 mm. The spectrophotometer recorded an integrated colour, the product of the reflectances of the different colours reflected in the same measurement and a direct function of the colour of minerals and grain size. The equipment (Figure 1) measured the reflectance of the illuminated measurement area, furnishing information on the percentage of reflectance received in each wavelength as a vector of 40 components (350 nm to 740 nm in intervals of 10 nm).

A total of 48 specimens with a surface area of 50 cm^2^, representative of three groups of 16 varieties of ornamental granite widely traded in the sector and different in terms of origin, colour and texture characteristics was used for data capture purposes.

Granite is characterized by a heterogenous surface in terms of colour and texture. In capturing colour by a contact measurement instrument such as a spectrophotometer—which spatially averages the light reflected from a fixed measurement area corresponding to the measurement aperture—measurements must be made at several points of the specimen to be able to assess the total real colour of the stone. The ideal approach would be to choose as small as possible a measurement area where the colour is representative of the overall rock colour (the result of the contributions of different minerals in different proportions), with the limitation, however, that the measurement area is determined by the measurement aperture used [2]. More specifically, following a series of tests, for this research we chose an aperture measuring 8 mm in diameter as the smallest aperture capable of characterizing the colour contributed by each of the minerals to the different granites evaluated.

A total of 160 colour measurements were randomly made of the 48 specimens, yielding a sample {*x_i_*, *y_i_*}, *i* = 1,2,...,160, where *x_i_* represented the 40 values for the rock spectrum in each wavelength band evaluated and *y_i_* was the class or variety of each granite specimen.

In our research, the number of measurements analysed for each rock surface was not in line with the recommendations of Prieto *et al*. [2], namely, 14 measurements for each 36 cm^2^ of rock surface when an 8-mm diameter measurement aperture is used. This was because granite varieties were not classified directly from the spectral information collected; rather, this information was processed by machine learning techniques for functional data, which, after suitable training and learning, acquired the ability to optimally solve classification problems with small samples. In particular, spectral information was collected for a set of 10 different measurement points, distributed randomly in the three available specimens, in order to classify each of the 16 types of granite to be identified. In the different measurements, each of the spectrophotometer sensors calculated the percentage of light reflectance in a strictly defined region of the visible-light wavelength range. Recorded data included, therefore, a great number of discrete variables.

Information processing was simplified by adopting a functional approach to the classification problem. Due to the nature of the information collected, considered a set of observations for a function in a finite set of values, it was necessary to perform a smoothing pre-process consisting of fitting the data to the nearest function representing them. This procedure simplified processing, with no loss of information, by reducing the number of state variables to 23.

The use of the Fourier orthonormal series as the basis functions in the smoothing pre-process (with **Φ** converted into an identity matrix) simplified the statistical treatment of the sample in terms of the number of state variables and also made the vectorial models functional.

The sample generated in the smoothing process can be represented as the set {**x***_i_*, *y_i_*} *i* = 1,2,..., 160 where **x***_i_* ∈ *R*^23^ indicates the spectral functions and *y_i_* the class of each specimen. The usefulness of a linear model in identifying the different types of granite was initially analysed for this functional set. Another model, also functional but more complex, was also built by implementing a SVM with a PUK kernel.

**Figure 2.**
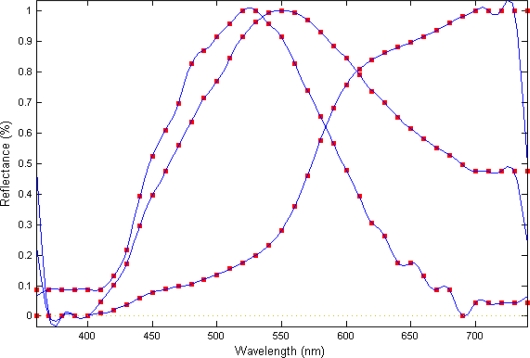
Reflectance curves resulting from the smoothing process for three granite specimens. Reflectance values are indicated as red squares.

Figure 2 shows the set of granite specimen reflectance values initially captured by the spectrophotometer and the resulting function after smoothing. Graphic representation of the spectral information complied for the 3 varieties evaluated clearly highlights differences in colour and tone.

To construct, compare and select the optimal algorithms for the processing of the spectral information, the sample was divided into a training set of 144 items—which underwent a process of cross-validation to determine optimal algorithm parameters—and a validation set of 16 randomly selected items representing each granite variety used to perform the final validation of the system.

For the 10-fold cross-validation, the entire training set was randomly divided into 10 disjoint sets; nine sets were used to train the model (for each range of variation in the internal parameters of the algorithm in question) and the remaining set was used to test the model. The optimal model resulting from the cross-validation process was selected on the basis of the average error rate for the 10 test sets generated in the process.

### 3.2. Expert System Integration in Granite Processing

**Figure 3.**
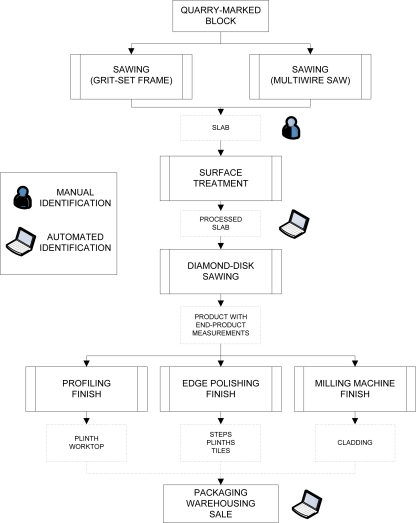
Flowchart depicting the granite production process and indicating phases where manual colour coding can be complemented by automated characterization.

**Figure 4.**
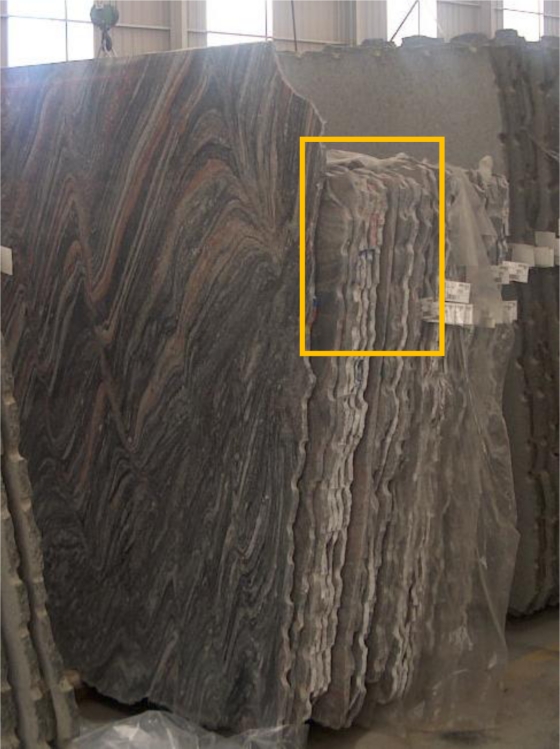
Granite slabs with manually applied colour codes (boxed area) used to identify slabs.

Optimal processing of the data collected makes no sense unless the automated characterization application can be implemented in the industrial granite production process. To do this, it was first necessary to identify the different phases where data would be captured and processed so as to be able to characterize, on an ongoing basis, the different rock types handled in a plant. Figure 3 depicts the different granite processing phases, indicating when slab edges are manually colour coded (Figure 4), and when the colour codes are lost (the diamond-disk sawing phase); it also shows the different phases where the granite could be characterized automatically.

The protocol for automatically identifying different granite types covers spectral information capture from each of the granite types to be characterized using a spectrophotometer and subsequent processing by the expert system on a laptop computer connected to the measuring equipment.

After the various possible treatment processes aimed at improving the visual appearance, texture and functionality of the stone (that is, polishing, bush-hammering, honing, flaming or sandblasting), initial spectral information for the slabs was collected that recorded the colour and brightness characteristics conferred on the rock by the different treatments. The expert system then directly classified the stone, provided suitable and enough spectral information was available to do so. If the granite type had not previously been characterized by the system, the learning process and optimal selection of model parameters would have to be readjusted. Note that in this first phase of automated identification the degree of adjustment of the system could be checked at any time against the information provided by manually applied colour codes, which effectively acted as an expert supervisor of the system.

Up to the cutting phase, slabs are clearly identified through the manual marking system. Hence, our methodology is a complementary method at this stage, redundant with respect to the traditional system but necessary to adjust, update and provide feedback to the automated classification system on the basis of reliable information (color codes), so as to ultimately be able to identify the lithology of each end product in subsequent processing stages when marks are lost.

The proposed expert system is particularly important in end-product identification and control at the packaging, storage and sale stages, as its potency, flexibility and portability is such that it enables proper management of the product in the final stages of the supply cycle.

## 4. Results and Discussion

To ensure optimum operation of the automated identification system and study how it could be practically applied in the granite industry, implemented and compared were the two FDA techniques—functional linear regression and SVMs for functional data, described in Sections 2.3 and 2.4 above—for resolving the problem of classifying the 16 granite varieties represented by 48 specimens. More specifically, analysed were the results obtained by statistically processing, using linear functional regression and a functional SVM (FVSM) with a PUK kernel, the functions obtained in the smoothing pre-process carried out on the original spectral information collected from the granite specimens.

A set of 100 basis functions was selected to perform the smoothing process, as this was the minimum number that provided a 99% fit between the 40 discrete points and the function.

The sample generated in the smoothing process, {*x_i_*, *y_i_*}, i = 1,2,…160, where each vector represented the spectral functions obtained, and where *y_i_* ∈ {1,2,..., 16} represented the class of each characterized sample, underwent a 10-fold cross validation process. The two best models were selected on the basis of the final mean error rate obtained for the test sets generated in the learning process. Table 1 shows, for each of the models implemented, the error rates obtained in the training and validation stages.

The poor functional linear regression results would indicate that the granite identification problem is not linear in nature, thereby justifying the use of non-linear and more complex functional machine learning techniques. The cross-validation methodology and the selection of a PUK kernel to implement the FSVM improved on the results obtained in previous research [4], where functional neural networks and FSVM with a Gaussian kernel were used.

The low error rate obtained using the FSVM model highlights the great predictive power of the algorithm, its flexibility and adaptability to the resolution of non-linear problems [11,23] and its ability to update to take account of new data without the initial structure of the model needing to be modified.

## 5. Conclusions

The correct identification of different types of granite in processing plants right through to the end-product stage optimizes the management and control of slabs and tiles and facilitates the implementation of new traceability systems in the sector.

The traditional color codes used to initially identify slabs are inevitably lost in subsequent processing phases. We have described a methodology that uses functional machine learning techniques to automatically classify rock at various stages of processing on the basis of spectral information captured by a spectrophotometer. Making the problem a functional one by smoothing the captured data enables all the information captured by the spectrophotometer to be analysed and evaluated and simplifies resolution of the granite classification or identification problem.

The good results obtained in processing spectral information using a FSVM with a PUK kernel would indicate this system to be an optimal model for inclusion, in the granite production process, as a system to automatically identify granite varieties. In addition to its great predictive power, the algorithm has great flexibility and is capable of updating to take new data into account.

At the industry level, it would be useful to implement a feedback and automatic application update procedure to make the adjustments necessary for the system to be able to correctly identify new varieties of stone and distinguish between stone types with similar mineralogical, textural and colour characteristics.

The main advantages of the proposed system are its functionality, flexibility and portability and the overall mixed-system approach to granite identification. The specific methodology developed in this research seeks to overcome the specific difficulties associated with the traditional method of manually marking slab edges for the purpose of characterization in subsequent processing stages. However, it complements, rather than overrides or replaces, this easy and rapid approach.

## Figures and Tables

**Table 1.** Mean training and validation error rates (percentage of poorly classified observations) for the two models.

	
	**ER train (%)**	**ER validation (%)**
**Functional Linear Regression**	15.35	26.43
**Functional SVM-PUK**	0	0.82
